# State Variations of Chronic Disease Risk Factors in Older Americans

**DOI:** 10.5888/pcd9.120143

**Published:** 2012-12-20

**Authors:** Stacey L. Tannenbaum, Diana Kachan, Cristina A. Fernandez, Laura A. McClure, William G. LeBlanc, Kristopher L. Arheart, David J. Lee

**Affiliations:** Author Affiliations: Diana Kachan, Cristina A. Fernandez, Laura A. McClure, William G. LeBlanc, Kristopher L. Arheart, David J. Lee, University of Miami Miller School of Medicine, Miami, Florida.

## Abstract

The objective of this study was to examine and compare 3 key health behaviors associated with chronic disease (ie, risky drinking, smoking, and sedentary lifestyle). We used data from the National Health Interview Survey from 1997 through 2010 to calculate the prevalence of these behaviors among older Americans and rank each state, and we analyzed overall trends in prevalence for each behavior over the 14 years. Older adults residing in Arkansas and Montana had the worst chronic disease risk profile compared with other states. These findings indicate the need for improved or increased targeted interventions in these states.

## Objective

Risky drinking, smoking, and sedentary lifestyle are key health behaviors associated with chronic disease and increased illness and death in older adults (1). Excessive drinking is associated with cancers of the liver, head and neck, colorectum, pancreas, and breast, as well as cardiovascular disease and diabetes (2). Smoking is associated with cancer and poor cardiovascular outcomes (1). Cardiovascular disease and cancer risk are increased by sedentary behavior (1). The objective of this study was to examine the prevalence and trends of these 3 health behaviors among older Americans and rank them at the state level to determine the best allocation of public health resources.

## Methods

Data were obtained from the National Health Interview Survey (NHIS), an annual, cross-sectional, multistage probability household survey of the noninstitutionalized civilian US population, from 1997 through 2010. Eligibility criteria were adults aged 65 or older (N = 79,973; representing 34,632,575 people). NHIS questions regarding the 3 variables are available online (3). Smoker was defined as “current smoker” (4). Risky drinking was defined as current drinkers having 10 or more drinks per week in men and 7 or more drinks per week in women, or having 5 or more drinks on 1 occasion, 1 or more times per year for men and women (4). Physical activity level was defined as compliance with the Healthy People 2010 goal of moderate physical activity for at least 30 minutes per day on 5 or more days per week or vigorous physical activity for at least 20 minutes per day on 3 or more days per week (5).

NHIS data were pooled and analyses were conducted using SAS version 9.2 (SAS Institute Inc, Cary, North Carolina), adjusting for sample weights and design effects (3). We calculated prevalence, standard errors (SEs), and 95% confidence intervals (CIs) and ranked states according to the prevalence of each risk factor indicator. We analyzed trends by using weighted linear regression of prevalence on year. Weight was generated with the inverse of the variance of prevalence. Some states were missing values because they did not meet the criteria for stable estimate analysis in all study years (6).

Because state-level data are not released to the public, all analyses were performed remotely at the National Center for Health Statistics Research Data Center. The study was approved by the University of Miami’s institutional review board.

## Results

The prevalence of smoking among US adults aged 65 years or older was 9.6% (Table 1). States with the highest smoking prevalence were Nevada (17.9%) and Kentucky (15.0%). States with the lowest rates of smoking were Utah (5.4%) and South Dakota (6.2%). Overall, 22% of older Americans reported risky drinking patterns; Arizona and New Hampshire had the highest prevalence, both at 29.0%, and the lowest prevalences were found in Kansas (14.4%) and Oklahoma (16.4%) (Table 2). Twenty-two percent of older Americans reported meeting physical activity recommendations; the highest prevalence was reported in Colorado (30.8%), Hawaii (34.8%), and Maine (40.1%), and the lowest prevalence was reported in Louisiana (13.4%), Mississippi (13.4%), and South Dakota (14.6%) (Table 3). Older Americans residing in Arkansas and Montana were in the top 10 worst rankings for all 3 behaviors.

**Table 1:** State-Specific Prevalence of Smoking **a** for Older US Adults: Pooled Data From the 1997–2010 National Health Interview Survey

State	N	Prevalence, % (95% CI)	SE	Rank^b^
All	79,973	9.6 (9.3–9.8)	0.1	NA
Alabama	1,536	9.4 (8.1–10.8)	0.7	23
Alaska^c^	73	5.3 (2.6–10.5)	1.9	1
Arizona	1,429	10.2 (7.8–13.1)	1.3	32
Arkansas	965	13.4 (10.3–17.3)	1.8	46
California	8,355	7.6 (6.9–8.3)	0.4	8
Colorado	927	10.2 (8.5–12.3)	1.0	32
Connecticut	1,037	7.5 (6.2–9.1)	0.8	6
District of Columbia^c^	205	9.1 (4.6–17.3)	3.1	20
Delaware	218	11.8 (7.5–17.9)	2.6	44
Florida	6,158	8.2 (7.2–9.3)	0.5	12
Georgia	1,863	10.0 (8.5–11.6)	0.8	29
Hawaii^c^	478	7.2 (3.6–13.8)	2.5	5
Idaho	309	10.7 (8.8–13.0)	1.1	35
Illinois	3,351	9.2 (8.1–10.4)	0.6	21
Indiana	1,697	13.4 (11.8–15.1)	0.9	46
Iowa	872	8.3 (6.9–9.9)	0.8	13
Kansas	794	11.2 (9.0–13.7)	1.2	42
Kentucky	1,151	15.0 (12.7–17.6)	1.3	50
Louisiana	1,189	11.0 (9.4–12.7)	0.9	40
Maine	458	10.0 (6.8–14.5)	2.0	29
Maryland	1,255	11.5 (9.2–14.2)	1.3	43
Massachusetts	1,847	9.9 (8.0–12.1)	1.0	27
Michigan	2,777	9.9 (8.8–11.1)	0.6	27
Minnesota	1,239	8.8 (7.4–10.3)	0.8	17
Mississippi	830	9.2 (6.7–12.4)	1.4	21
Missouri	1,771	10.9 (9.1–13.0)	1.0	38
Montana	306	13.9 (10.2–18.9)	2.2	49
Nebraska	613	7.5 (5.6–10.0)	1.1	6
Nevada	474	17.9 (14.1–22.5)	2.1	51
New Hampshire^c^	322	11.1 (5.8–20.2)	3.5	41
New Jersey	2,467	8.9 (7.8–10.3)	0.6	18
New Mexico	767	12.2 (8.8–16.7)	2.0	45
New York	5,460	8.6 (7.8–9.5)	0.4	16
North Carolina	2,303	9.0 (7.9–10.2)	0.6	19
North Dakota	263	8.1 (5.9–11.0)	1.3	11
Ohio	3,343	10.2 (8.9–11.7)	0.7	32
Oklahoma	1,033	10.9 (8.9–13.2)	1.1	38
Oregon	996	7.7 (6.3–9.5)	0.8	9
Pennsylvania	3,765	9.7 (8.6–10.8)	0.5	26
Rhode Island^c^	261	7.0 (3.7–13.0)	2.3	4
South Carolina	1,225	9.6 (8.7–10.6)	0.5	24
South Dakota	293	6.2 (3.7–10.2)	1.6	3
Tennessee	1,517	10.8 (8.6–13.5)	1.2	36
Texas	5,196	10.8 (9.9–11.8)	0.5	36
Utah	522	5.4 (3.4–8.4)	1.3	2
Vermont	149	7.7 (4.9–11.7)	1.7	9
Virginia	1,994	10.0 (8.6–11.7)	0.8	29
Washington	1,441	9.6 (7.7–11.9)	1.1	24
West Virginia	579	8.4 (6.5–10.9)	1.1	14
Wisconsin	1,737	8.5 (7.1–10.1)	0.8	15
Wyoming	163	13.7 (9.9–18.6)	2.2	48

Abbreviations: CI, confidence interval; SE, standard error; NA, not applicable.

a Defined as current smoker.

b States with the same ranking indicate identical prevalence estimates; in these instances, the subsequent state skips a number in ranking and continues.

c Prevalence estimate considered statistically unreliable with a relative SE of 30% or more or sample size of fewer than 50 (6).

**Table 2:** State-Specific Prevalence of Risky Drinking **a** for Older US Adults: Pooled Data From the 1997–2010 National Health Interview Survey

State	N	Prevalence, % (95% CI)	SE	Rank^b^
All	31,432	22.0 (21.4–22.6)	0.3	NA
Alabama	324	19.8 (14.8–25.9)	2.8	18
Alaska^c^	<50	29.9 (11.8–57.6)	12.4	51
Arizona	677	29.0 (25.3–32.9)	1.9	49
Arkansas	197	26.1 (19.3–34.4)	3.9	42
California	3,702	23.8 (21.9–25.9)	1.0	35
Colorado	453	21.4 (17.5–25.8)	2.1	26
Connecticut	534	17.4 (14.5–20.7)	1.6	6
District of Columbia^c^	81	20.6 (10.0–37.4)	7.0	22
Delaware	110	16.9 (12.0–23.4)	2.9	5
Florida	2,947	24.1 (22.3–25.9)	0.9	38
Georgia	497	18.5 (14.7–23.0)	2.1	9
Hawaii	145	27.2 (18.0–38.8)	5.3	45
Idaho	114	23.0 (13.6–36.1)	5.8	32
Illinois	1,362	22.6 (19.6–26.0)	1.6	30
Indiana	517	23.9 (18.7–30.1)	2.9	37
Iowa	403	22.6 (18.1–27.9)	2.5	30
Kansas	266	14.4 (9.5–21.3)	3.0	2
Kentucky	241	18.8 (12.4–27.5)	3.8	13
Louisiana	339	25.3 (18.9–33.0)	3.6	41
Maine	220	27.3 (21.7–33.7)	3.0	47
Maryland	527	20.8 (17.0–25.2)	2.1	24
Massachusetts	917	23.3 (19.1–28.2)	2.3	34
Michigan	1,209	22.1 (19.5–24.9)	1.4	28
Minnesota	658	21.2 (18.3–24.3)	1.5	25
Mississippi	163	18.5 (12.9–26.0)	3.3	9
Missouri	634	16.7 (13.2–20.9)	2.0	4
Montana	180	27.2 (19.1–37.3)	4.7	45
Nebraska	266	18.6 (15.6–21.9)	1.6	12
Nevada	239	20.2 (15.2–26.2)	2.8	19
New Hampshire	167	29.0 (20.7–39.0)	4.7	49
New Jersey	1,144	18.8 (16.3–21.5)	1.3	13
New Mexico	279	23.8 (19.5–28.8)	2.4	35
New York	2,341	20.7 (19.1–22.5)	0.9	23
North Carolina	531	22.5 (17.5–28.3)	2.8	29
North Dakota^c^	115	13.9 (6.2–28.2)	5.4	1
Ohio	1,154	19.3 (16.8–22.1)	1.3	15
Oklahoma	257	16.4 (11.7–22.5)	2.8	3
Oregon	491	26.9 (21.6–32.9)	2.9	44
Pennsylvania	1,682	18.5 (16.0–21.3)	1.4	9
Rhode Island	131	24.9 (17.4–34.4)	4.4	40
South Carolina	332	21.8 (18.1–25.9)	2.0	27
South Dakota	106	18.2 (11.6–27.5)	4.0	8
Tennessee	374	19.5 (14.6–25.7)	2.8	16
Texas	1,598	26.2 (23.6–28.9)	1.4	43
Utah	132	20.4 (13.7–29.3)	4.0	21
Vermont	86	20.3 (18.5–22.2)	0.9	20
Virginia	664	24.6 (20.9–28.7)	2.0	39
Washington	722	23.0 (19.0–27.5)	2.2	32
West Virginia	113	19.7 (14.1–27.0)	3.3	17
Wisconsin	988	17.8 (16.1–19.7)	0.9	7
Wyoming	70	28.0 (18.8–39.5)	5.3	48

Abbreviations: CI, confidence interval; SE, standard error; NA, not applicable.

a Defined as 10 or more drinks per week in men and 7 or more drinks per week in women, or 5 or more drinks on 1 occasion 1 or more times per year for both men and women.

b States with the same ranking indicate identical prevalence estimates; in these instances, the subsequent state skips a number in ranking and continues.

c Prevalence estimate considered statistically unreliable with a relative SE of 30% or more or sample size of fewer than 50 (6).

**Table 3:** State-Specific Prevalence of Meeting Physical Activity Recommendations **a** for Older US Adults: Pooled Data From the 1997–2010 National Health Interview Survey

State	N	Prevalence, % (95% CI)	SE	Rank^b^
All	74,470	22.0 (21.6–22.5)	0.2	NA
Alabama	1,427	19.9 (17.1–22.9)	1.5	32
Alaska	62	20.9 (14.6–29.0)	3.7	29
Arizona	1,340	27.0 (24.8–29.3)	1.1	8
Arkansas	908	15.1 (10.4–21.4)	2.8	47
California	7,839	26.9 (25.4–28.3)	0.7	11
Colorado	876	30.8 (26.7–35.3)	2.2	3
Connecticut	966	26.3 (22.0–31.2)	2.4	14
District of Columbia	191	24.0 (17.5–40.4)	4.6	19
Delaware	206	27.5 (16.2–34.1)	5.9	7
Florida	5,814	26.6 (24.2–29.1)	1.2	12
Georgia	1,743	16.3 (14.3–18.5)	1.1	45
Hawaii	457	34.8 (27.5–42.9)	4.0	2
Idaho	290	22.7 (18.6–27.5)	2.3	24
Illinois	3,103	21.4 (19.6–23.4)	1.0	28
Indiana	1,585	15.1 (12.8–17.6)	1.2	47
Iowa	820	25.1 (22.5–28.0)	1.4	15
Kansas	749	23.5 (19.2–28.4)	2.3	21
Kentucky	1,062	15.5 (13.1–18.4)	1.4	46
Louisiana	1,120	13.4 (10.0–17.7)	2.0	50
Maine	436	40.1 (33.0–47.6)	3.7	1
Maryland	1,176	22.8 (20.0–25.7)	1.5	23
Massachusetts	1,727	22.3 (19.2–25.7)	1.6	26
Michigan	2,535	22.9 (20.6–25.5)	1.3	22
Minnesota	1,153	27.6 (25.2–30.1)	1.3	6
Mississippi	763	13.4 (8.8–19.8)	2.8	50
Missouri	1,648	19.6 (17.2–22.3)	1.3	35
Montana	284	17.7 (14.2–21.8)	2.0	43
Nebraska	578	20.5 (14.5–28.2)	3.5	30
Nevada	449	22.6 (18.1–27.9)	2.5	25
New Hampshire	305	27.0 (19.7–35.7)	4.1	8
New Jersey	2,293	18.2 (15.9–20.7)	1.2	38
New Mexico	723	23.7 (19.3–28.8)	2.4	20
New York	4,970	19.7 (18.3–21.2)	0.7	34
North Carolina	2,204	18.7 (16.3–21.3)	1.3	37
North Dakota	246	19.5 (14.8–25.1)	2.6	36
Ohio	3,051	17.7 (16.0–19.5)	0.9	43
Oklahoma	937	18.2 (13.8–23.5)	2.5	38
Oregon	870	26.4 (22.1–31.3)	2.3	13
Pennsylvania	3,433	19.9 (18.0–21.5)	0.8	32
Rhode Island	246	21.6 (16.2–28.2)	3.0	27
South Carolina	1,163	17.8 (14.5–21.6)	1.8	42
South Dakota	279	14.6 (9.6–21.7)	3.0	49
Tennessee	1,420	18.1 (16.0–20.4)	1.1	40
Texas	4,916	20.3 (18.7–21.9)	0.8	31
Utah	496	28.5 (23.2–34.4)	2.9	5
Vermont	134	27.0 (17.7–38.9)	5.5	8
Virginia	1,849	24.2 (22.0–26.6)	1.2	18
Washington	1,338	29.8 (26.7–33.0)	1.6	4
West Virginia	519	17.9 (14.0–22.5)	2.2	41
Wisconsin	1,617	25.0 (23.0–27.1)	1.0	16
Wyoming	154	24.9 (16.8–35.3)	4.7	17

Abbreviations: CI, confidence interval; SE, standard error; NA, not applicable.

a Moderate physical activity for at least 30 minutes per day on 5 or more days per week or vigorous physical activity for at least 20 minutes per day on 3 or more days per week.

b States with the same ranking indicate identical prevalence estimates; in these instances, the subsequent state skips a number in ranking and continues.

A downward trend in smoking was observed during the 14 years for California (slope, −0.32; SE, 0.09; *P* = .004) and South Carolina (slope, −0.54; SE, 0.21; *P* = .046), and an increased trend for risky drinking was observed in Massachusetts (slope, 1.07; SE, 0.39; *P* = .026). In North Carolina (slope, 0.82; SE, 0.25; *P* = .007) and Texas (slope, 0.57; SE, 0.16; *P* = .004), an upward trend in exercise compliance was observed. Trend analysis was not conducted for 7 states and the District of Columbia due to insufficient sample sizes.

## Discussion

The average age of Americans is expected to increase substantially in the coming years (7). Modifying key health behaviors and creating cost-effective interventions may contribute to decreasing illness and death in this growing population demographic (8).

Lifestyle changes that occur with aging can affect chronic disease risk. Older adults who exercise regularly have a reduced mortality risk (9), but those who drink alcohol excessively are more prone to oxidative stress, which further increases the incidence of chronic disease (10). A twofold higher mortality rate was shown for older male smokers than nonsmokers (11). Risky drinking with aging has been positively associated with vigorous physical activity and negatively associated with current smoking, possibly reflecting better health among adults who engage in risky drinking as they age (12). Nevertheless, excessive alcohol consumption is associated with risk of falls (1) and adverse medication interactions in older Americans (10).

Limitations of this study included an inability to use estimates from all states due to small sample sizes or unstable estimates in some states (ie, a relative SE of ≥30%). We were unable to conduct complete trend analyses for all states given sample size limitations. The strength of this study was the access to a large set of sample data at the state level for prevalence comparisons in older Americans.

Public health resources should focus on specific interventions to affect behaviors in states with residents at high risk for developing chronic disease. These resources can include a purposeful combination of the following: 1) increasing tobacco excise taxes, proven to be the most effective means to decrease smoking (1), 2) using online and telephone substance abuse treatment facility locators and media campaigns to disseminate information on alcohol abuse (1), and 3) enhancing access to recreational and physical activity facilities in communities specific to older Americans, pursuant to the Healthy People 2010 guidelines (5). Emphasis on geographic aggregation of risk factors should be considered so that integrated and tailored prevention activities can be developed and customized to each state’s profile and funds be made appropriately available. States with the highest prevalence of 2 or 3 risky behaviors should review resource allocation to promote health more effectively.
